# A tubular electrode assembly reactor for enhanced electrochemical wastewater treatment with a Magnéli-phase titanium suboxide (M-TiSO) anode and *in situ* utilization†

**DOI:** 10.1039/d1ra02236a

**Published:** 2021-07-20

**Authors:** Jiabin Liang, Shijie You, Yixing Yuan, Yuan Yuan

**Affiliations:** State Key Laboratory of Urban Water Resource and Environment, School of Environment, Harbin Institute of Technology Harbin P. R. China Liangjiabin1234@foxmail.com; School of Biological Engineering, Beijing Polytechnic Beijing 100176 P.R. China

## Abstract

The electrochemical oxidation technology has been widely used for the waste water treatment and water reuse because of its easy-to-operate nature, an effective removal of pollutants and non-secondary pollution. However, the price of electrode materials, the limitation of mass transfer and the associated effects on contaminant degradation hamper its application. Within this context, an *in situ* utilization tubular electrode assembly reactor (TEAR) was proposed, in which a stainless steel pipe (SSP) was used as the cathode, and a tubular Magnéli-phase titanium suboxide (M-TiSO) anode was posited in the center of that pipe. Besides the cathode and anode, an integral electrochemical system to treat water pollutants was constituted with a spiral static mixer made from three-dimensional (3D) printing. A spiral static mixer was pushed into the interspace of electrodes to minimize the adverse effect caused by inhomogeneous distribution of pollutants. Here, the effects of current density and resident time on the removal of methylene blue (MB) and total organic carbon (TOC) were investigated, the corresponding hydrodynamics was studied using computational fluid dynamics (CFD), and the long-term stability of removing MB by the reactor was discussed. The results indicated that the MB and TOC removal rate was enhanced at specific current density with a static mixer and the velocity distribution tended to be more homogeneous. Moreover, the anode surface shear force and heat transfer were increased by improving the fluid state. This study proposed an *in situ* utilization concept and provided a potential value for feasible and efficient water treatment.

## Abstract 2

A stainless steel pipe (SSP) was used as a cathode. A tubular Magnéli-phase titanium suboxide (M-TiSO) anode was posited in the center. A spiral static mixer was used to process intensification.

## Graphical abstract

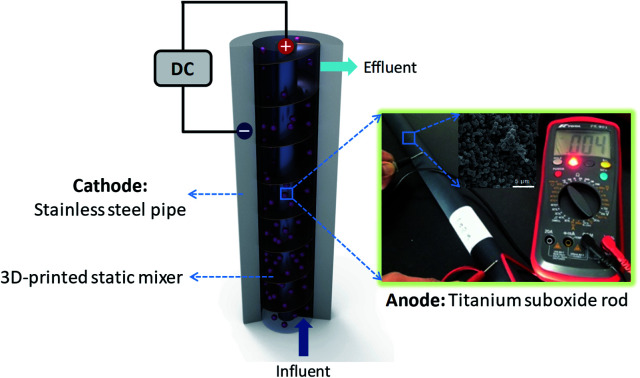

## Introduction

Environmental issues are gradually becoming the focus of human concern with regard to the unsustainable development on natural resources and environment. Nowadays, technologies, especially electrochemical oxidation (EO), which has been gaining an increasing popularity by virtue of its high efficiency, simplicity, sustainability, and ease of manipulation, have been increasingly exploited and imposed to treat pollutants.^1,2^ Tremendous potential offered by electrochemistry has been realized in a broad swath of environmental applications such as the removal of phenols, drugs, pesticides, antibiotics and algae.^3,4^ However, there remain several aspects of EO that need further investigation if the engineered applications are to be better implemented and developed.

Two approaches were focused for promoting the operation efficiency of the EO process. The first one mainly focuses on the electrode materials with high conductivity, activity and stability, while the second one is referred to as process intensification (PI).^5,6^ PI is regarded as one of the most important progresses in modern chemical engineering, and in the EO process, it should be related to the intensification of several factors such as electrode materials, electrolytic cell design, operation, electrolytes, as well as mass and heat transfer.^7–9^ However, owing to electrochemical reactions, the PI of EO is more challenging than that of conventional thermal catalysis, particularly in the case of mass transfer and scale-up issues. For example, to promote the removal of organic pollutants, EO is often operated under diffusion control, where the current density is larger than the limiting current density. The mass transfer limitation can be mitigated by modifying hydrodynamic conditions through proper design and operation of electrolytic cells, such as increasing electrode pores or interspaces to increase the turbulence condition, or increasing flow rates to improve the contact between organic pollutants and the electrode.^10,11^ However, these methods are carried out at the cost of either decreasing the treating capacity or increasing energy consumption.

In a practical point of view, the PI of the EO process should aim to design an electro-catalytic cell with electrodes that are conductive, stable, and active for ˙OH radical production. The electrodes should require a small space that allows continuous on-line reactor design and performance at minimum energy consumption, which made it easy for operation and maintenance. In addition, the reactor needs to be optimized for the maximization of waste water capacity and minimization of undesired by-products (*e.g.* gas bubbles and heat).^7,12,13^

**Fig. 1:**
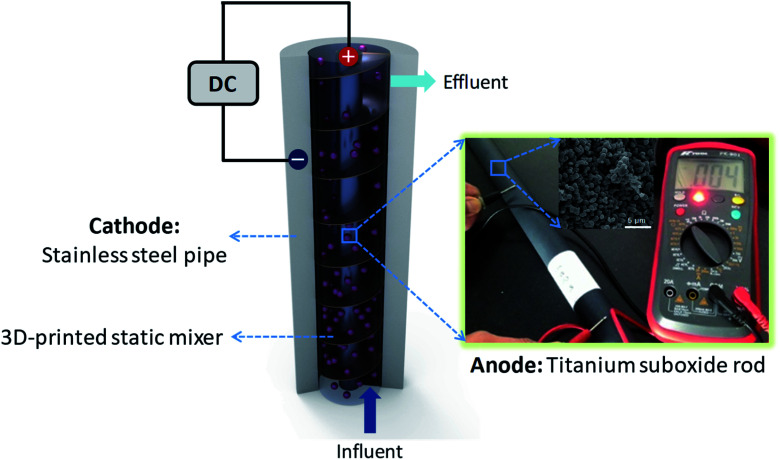
Schematic of TEAR (DC: direct current).

To achieve these goals, we herein report for a continuously operational *in situ* utilization tubular electrode assembly reactor (TEAR) constructed upon the Magnéli-phase titanium suboxide (M-TiSO) anode, a stainless steel pipe (SSP) cathode and a static mixer (Fig. 1). The TEAR *in situ* utilization ideology was proposed by taking into account several factors including the selection of the M-TiSO anode that originates from its properties of great conductivity and corrosion resistance,^14–16^ the application of SSP as a supplier for water transportation without concerning the occurrence of electrochemical corrosion and the adoption of a static mixer for strengthening the hydrodynamic property. Moreover, the long-term stability of the TEAR for MB removal was investigated, demonstrating that the M-TiSO exhibited excellent stability during electrochemical oxidation and the M-TiSO anode was superior for efficient MB removal, with less oxygen evolution (side reactions) and better current efficiency during electrolysis.

## Materials and methods

All chemicals used here were of analytical reagent grade, and all experiments were conducted with at least two parallel samples at ambient temperature and standard atmospheric pressure.

### Design and configuration of the TEAR

The tubular electrode assembly reactor (TEAR) was constructed using a 7 cm diameter and 35 cm high cylindrical SSP (2 mm in thickness) with a total volume of 1196.8 mL. Two ports were created on the top and bottom of the reactor to collect the effluent and influent stream, respectively. The outer stainless steel wall of the reactor was *in situ* used as the cathode, and the TiSO rod (obtained from Ti-Dynamics Co. Ltd. China) with a length of 35 cm and a diameter of 2 cm (surface area of 219.8 cm^2^) was situated in the center of the SSP cathode and used as the anode. The Plexiglas Kenics static mixer produced by 3D printing was suited tightly between the electrodes, resulting in a wet volume of 1042.9 mL. Two alligator clips were individually used for ohmic contact of both electrodes, which were connected with DC-regulated power supply (0–35 V and 0–3.5 A; TP-3003D, TeKPower, China) by copper wires. The design and configuration of the TEAR is schematically illustrated in Fig. 1.

### Operation

The azo dye (*i.e.* methylene blue, MB) of analytical purity was used as a synthetic dye pollutant without further purification. The synthetic wastewater contained a specific concentration of MB (50 mg L^−1^) and Na_2_SO_4_ (0.5 mol L^−1^) as supporting electrolytes. The solution was prepared by using deionized (DI) water to minimize the interferences. The continuous decolorization of MB by EO in the TEAR was performed under galvanostatic conditions by applying current density in the range of 0.8–15 mA cm^−2^. The feeding wastewater was continuously supplied into the TEAR at a flow rate from 6.95 to 34.76 mL min^−1^. To examine the stability of electro-oxidation, long-term decolorization efficiency and periodic CV tests were recorded by operating the TEAR for multicycles under defined conditions.

### Analyses and calculations

The synthetic wastewater sample was collected for analysis after the reactor was operated stably for minimum time of 2 h. The decolorization of MB was assessed by measuring the absorbance at 664 nm in the UV-Vis spectrum using a UV-2550 spectrophotometer (Shimadzu, Japan). The mineralization of MB was evaluated by measuring total organic carbon (TOC) using a TOC analyzer (VSCN8, Shimadzu, Japan). The electrochemical tests were conducted in 0.5 mol L^−1^ Na_2_SO_4_ with 50 mg L^−1^ MB using a dual working electrode PARSTAT (CHI750D, Chenhua Co. Ltd.) electrochemical system at room temperature (25 °C). Besides, the reactor design and constitution were done using the AutoCAD2016 software and then the corresponding structure was meshed with approximately 10 million cells using an ICEM processor. The computation was carried out with a three-dimensional single precision, which produced accurate predictions in many cases.^17–19^ The maximum residual tolerance was set as 10^−4^ for classical calculating equations. The ambient water was adopted as the fluid and the inlet boundary velocity was set as 2 cm s^−1^ according to the actual situation. The pressure-outlet boundary and solid-wall boundary were assumed for the outlet and electrodes, respectively. The anode was chosen as a heat resource with a thermal flux of 4 kW m^−2^ for temperature field simulation. The physical properties of electrolytes for simulation are listed in Table S1.†

The mass continuity can be written as follows:1
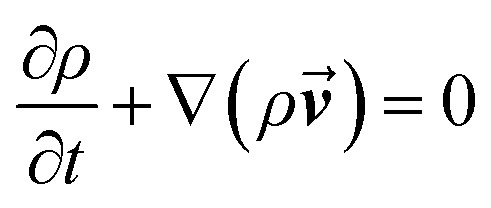
where 
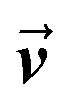
 is the velocity and *ρ* is the density of the liquid.

The momentum can be written as follows:2
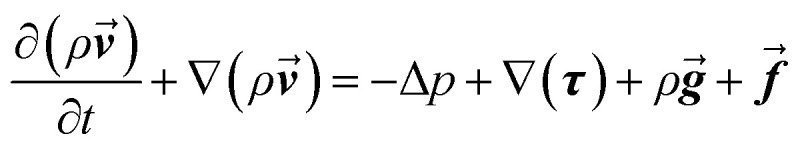
where 
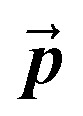
 is the pressure, 
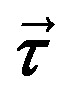
 is the stress tensor, 
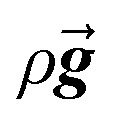
 is the gravitation body force, and 
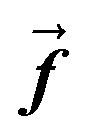
 is the external force vector.

The energy can be written as follows:3
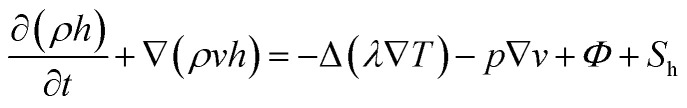
where *h* is the sensible enthalpy, *λ* is the thermal conductivity, *T* is the temperature, *Φ* is the viscous heat of liquid, and *S*_h_ is volumetric heat source.

## Results and discussion

### Configuration and composition of the TEAR

**Fig. 2:**
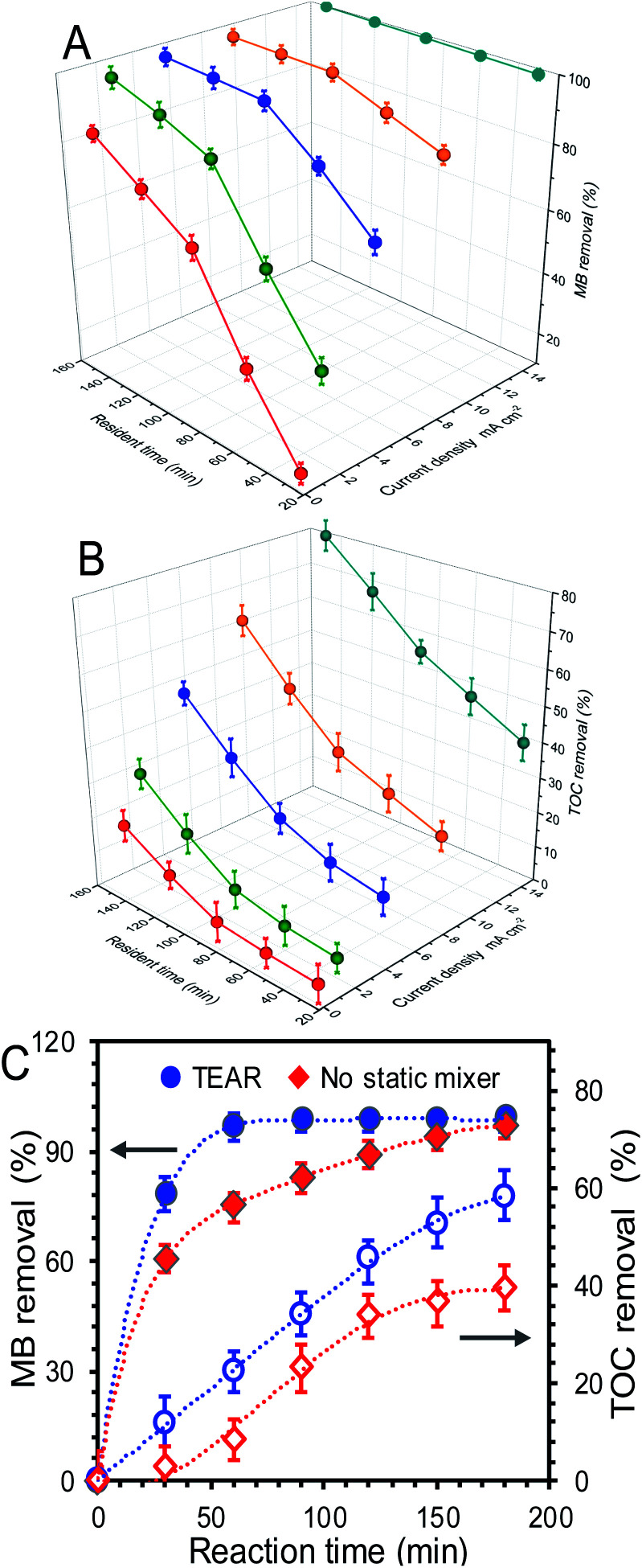
Effect of current density and resident time on (A) MB and (B) TOC removal. (C) The corresponding removal in TEAR and the reactor without static mixer at a current density of 9 mA cm^−1^ and a resident time of 150 min. The error bars ± S.D. represent the measurement in triplicate.

As stated above, the TEAR was constructed by a SSP cathode and a tubular M-TiSO anode to remove the pollutant by electro-oxidation, in which the abatement efficiency depends to a great extent on the anode materials. According to XRD and SEM analyses, the characteristic peaks can be indexed to the mixture of M-TiSOs (*i.e.* Ti_4_O_7_, Ti_5_O_9_, Ti_9_O_17_, MDI Jade5.0) with approximately 0.5–2 μm spherical morphology and 0.2–2 μm macroporous structure that originated from particle packing (Fig. 2). As reported, the electrocatalytic anode material and reactor configuration play an extreme important role in water treatment.^20,21^ Here, the reactor was designed through an *in situ* assembly ideology and the M-TiSO anode material, which were selected for the following properties: first, M-TiSOs exhibits excellent electrical conductivity (900–1050 S cm^−1^), physical and chemical stability, high oxygen evolution potential (+2.6 V *vs.* SHE), high catalytic activity and selectivity. M-TiSOs is a kind of material resistant to erosion, corrosion and formation of passive layers, and it has the superiority of low cost and long service life (30 years).^22,23^ Second, this material can be produced into any geometrical shape of interest and the M-TiSO anode was easily produced *in situ* in the middle of the tubular reactor like a bar, which made the pollutions contact the anode surface sufficiently. Third, SSP is widely used as a supplier for water transportation, and its intrinsic electrical conductivity well matches the requirement of cathodes with no need to consider the electrochemical corrosion issues. This *in situ* utilized strategy pushes the EO development on an applicable scale. Last, static mixer is used here for strengthening the hydrodynamic property within the region adjacent to the electrode surface. The static mixer is a motionless insert installed in pipes, channels or ducts, which can redistribute streamlines at different scales in radial and tangential directions transverse to the main flow. Thus, the mass transfer can be improved easily by using only the kinetic energy of flowing fluid without extra energy input through the *in situ* static mixer. The enhanced mass transfer will be helpful to mitigate the problems associated with electrode fouling, gas bubble accumulation and heat dissipation.

### Effect of current density and hydrodynamic conditions

The performance of the TEAR for the removal of the dye material (MB) under continuous operation in the presence of a static mixer was investigated. As electro-oxidation proceeded, the blue synthetic wastewater gradually changed into transparent, indicating the effective action of electrogenerated ˙OH radicals on fragmentation of the dye chromophoric groups in MB. In this context, the abatement efficiency of MB was positively dependent on the supplied resident time (mainly related to reaction time in this continuous non-reflow operation) and current density. Current density as the most frequently specified parameter can control the reaction rate, which commonly defines the efficiency of the process. As shown in Fig. 2, with the increase in resident time and current density in the range of 30–150 min and 0.8–15 mA cm^−2^, respectively, the removal rates of MB and TOC showed different increase degrees at the end of 3 h of treatment. According to the constructed response surface, the removal rate optimized at 9 mA cm^−2^ with a resident time of 90 min can obtain the final MB and TOC removals of 98.35% and 35.51% in the treated effluent, respectively. It was noted that a further increase in current density and resident time did not necessarily enhance the oxidation efficiency. A further increase in current density to 15 mA cm^−2^ and resident time to 120–150 min did not contribute significant improvement of MB removal (Fig. 2A), instead, caused a sharp TOC decline and far more energy consumption (Fig. 2B). Such phenomena indicated that the chromophoric groups can be easily destroyed while the TOC removal required further oxidation. Moreover, the MB removal exhibited a sharp increase when the current density and resident time were lower than 5 mA cm^−2^ and 90 min respectively and then trended gently after that, and the negative influence of the rising current density during the EO treatment of MB could be attributed to the occurrence of the OER and/or mass transport limitations. However, the TOC removals were totally opposite, wherein the TOC removals increased faster when the current density and resident time were higher than 5 mA cm^−2^ and 90 min, respectively, indicating that more energy and intensive oxygen species were needed for further mineralization of MB. More significantly, the electro-oxidation of MB was more efficient and clean compared with other treatments (adsorption, flocculation, biotechnology, *etc.*), which may cause secondary contamination with the extra chemicals input.^24,25^ Besides, the anode material of M-TiSOs used here can dramatically improve the degradation efficiency due to its unique properties specified as follows: (i) excellent conductivity and stability, (ii) large working potential window, especially high oxygen evolution potential (+2.6 V *vs.* SHE), implying less oxygen evolution (side reactions) and better current efficiency during electrolysis. The M-TiSOs was also reported to be more efficient and cost-effective than other materials for the destruction of dyes, phenol, and some other organic pollutants.^26–28^

In order to investigate the effect of the static mixer on MB and TOC removal, the reactor without any mixer was conducted as a comparative study. As shown in Fig. 2C, the decolorization was fast proceeded within 30 min and then experienced a slow decomposition course until it reached ∼100% removal. For the TEAR, the maximum abatement efficiency of 97.1% can be achieved compared to 75.36% for the reactor without any mixer at 60 min, and it took 60 minutes for the TEAR to reach the maximum removal value (99.3%) while took 180 minutes for the reactor without any mixer (97.0%), demonstrating that the abatement efficiency can be greatly enhanced by improving the mass transfer condition. However, the TOC removal exhibited a continuous increase with the reaction time and can reach 58.1% and 39.6% for the TEAR and the reactor without any mixer, respectively. This value was comparative with a series of extensive studies on reactor configuration and anode materials.^16,29–31^ Besides, the TOC removal increased much slower for the control reactor than for the TEAR at the end of 3 h, indicating that the TEAR had a great potential to continue enhancing the TOC removal.

### Long-term stability of the TEAR

**Fig. 3:**
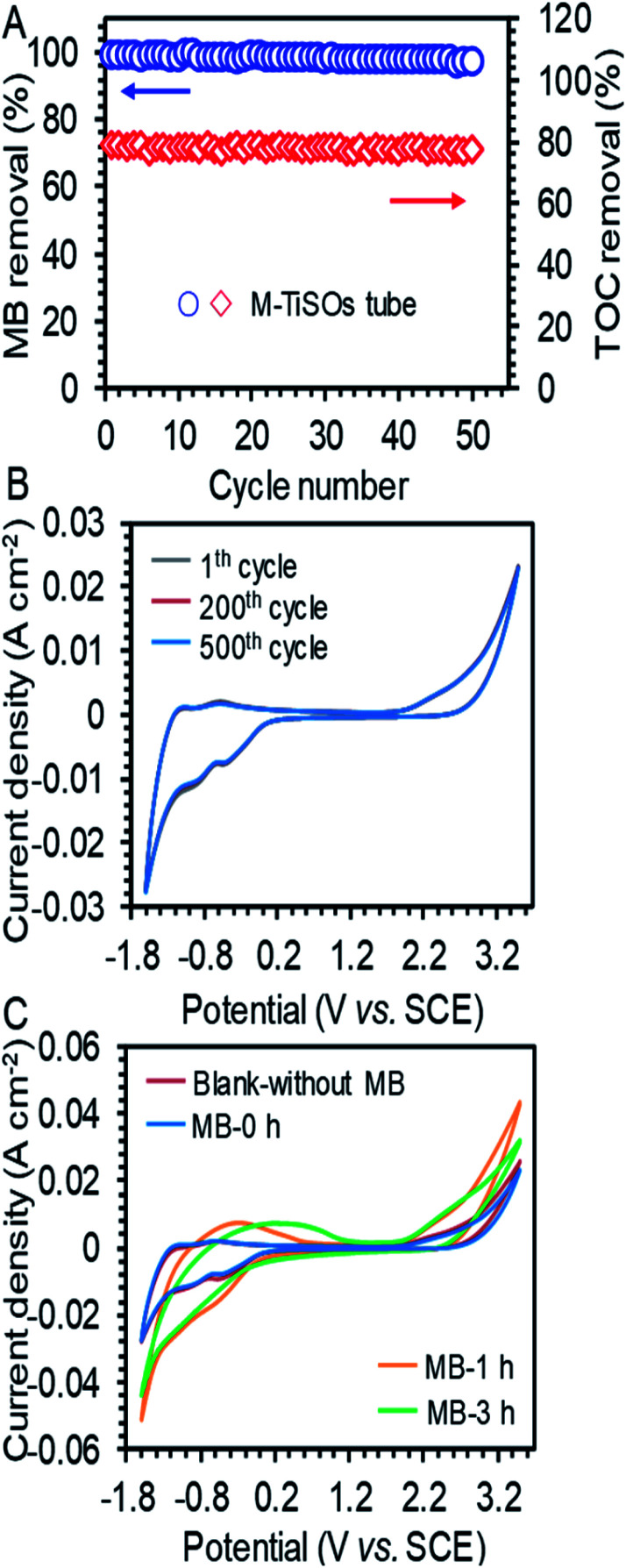
(A) Stability performance of MB and TOC removal during 50 cycles of operation and (B) periodic CV tests in 0.5 mol L^−1^ Na_2_SO_4_ with 50 mg L^−1^ MB solutions at a scan rate of 50 mV s^−1^ for the M-TiSO electrode. (C) CV curves in blank (without MB), untreated (0 h), and treated solutions (1 and 3 h) at a scan rate of 50 mV s^−1^.

The long-term stability of the TEAR for MB removal was investigated by conducting periodic experiments under a high current density of 15 mA cm^−2^. As shown in Fig. 3A, the maximum MB removal of 89.2% and TOC removal of 79.04% exhibited no obvious decline, showing a superior stability of the TEAR with the M-TiSOs anode for long-term decomposition of MB. The degradation performance under higher current density may be associated with the deactivation of the outside surface of the M-TiSOs thin film, leading to the formation of more oxidative titanium oxides.^32^ Besides, the multiturn CV curves were performed in the MB solution with the potential range from −1.6 to + 3.5 V *vs.* SCE. The M-TiSOs electrode survived 500 cycles without significant loss in current responses (Fig. 3B), demonstrating that the M-TiSOs exhibited excellent stability during electrochemical oxidation, implying a remarkable potential for pollutant degradation. Based on the above-mentioned analyses and reported research, the M-TiSO electrode exhibited superior stability under many conditions including in strong alkaline, acid, pH-neutral, bacteria-rich, high temperature, and high current density conditions,^23,33–35^ further implying a stable application in the electrochemical system. Moreover, according to the thermodynamics of oxygen defective in Magnéli phases, the spin polarization lowered the normalized defect formation energies marginally. For Ti_4_O_7_ and Ti_5_O_9_, the lowering was of the order of 0.06 eV, which did not affect the stability ordering of these Magnéli phases, resulting in a relative stable crystal structure for M-TiSOs.^36–38^ As shown in Fig. 3C, the M-TiSOs exhibited a high oxygen evolution potential of + 2.0 V *vs.* SCE at a current density of 4 mA cm^−2^ in both MB-containing and non-MB Na_2_SO_4_ solution (blank), a value consistent with the literature,^39^ indicating less side reactions and more efficient performances in pollutant removal. Besides, there are no obvious difference in blank and MB solutions, demonstrating that the MB removal was achieved *via* indirect electrochemical oxidation and mediated by ˙OH radicals. It can also be observed that a very slightly decrease in OER current density can be obtained after added MB, which may be caused by the block of OER active sites by MB. After continuous electrolysis, the M-TiSO surface progressively changed, showing totally different CV transformation. For the suspensions treated for 1 h, the color had changed from dark blue to super light-colored, which was in accordance with electro-oxidation decolorization of the chromophoric groups. As electrolysis proceeded for 3 h, the color had become transparent, which was in line with the process of further removing the TOC. Besides, the current response in 6 h treatment had been decreased due to the further degradation of existing chemicals, which was in agreement with the actual experiment. With these analyses, it was thus inferred that the M-TiSO anode was superior for efficient MB removal, with less oxygen evolution (side reactions) and better current efficiency during electrolysis.

### Mechanisms of enhanced performances of the TEAR

As reported, mass transfer (MT) was a crucial factor that determined the abatement efficiency in the EO system.^40^ The virtues of an enhanced MT were to improve the below aspects. First, strengthening the mass contact with the electrode enhances the reaction rate, because the reaction occurred on the surface of the electrode. Second, eliminating the adverse effect of the inevitably produced and adsorbed bubbles on the electrode surface during electrolysis can decrease the efficiency by blocking the reaction sites and causing catalyst stripping. Last, minimizing the heat influences the current efficiency and scale-up reactors. A proper design of reactor can be helpful to diminish the above-mentioned issues, for example, pushing the static mixer into the water pipe was prospected to improve the mass transfer conditions.

**Fig. 4:**
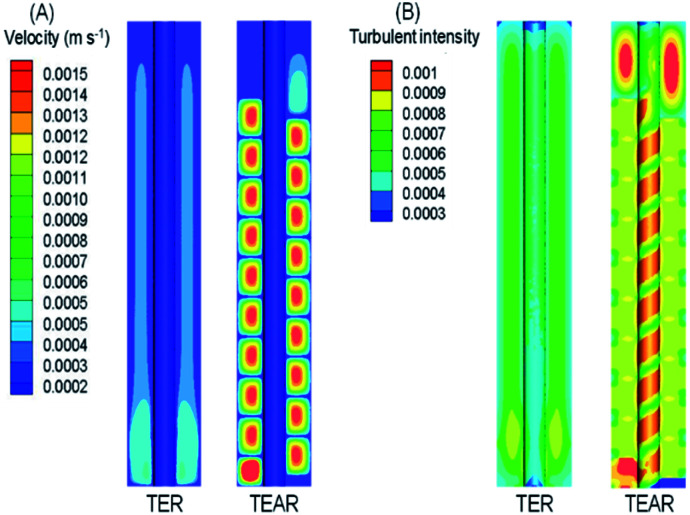
Front view of distribution of (A) velocity and (B) turbulent intensity in the TER and TEAR under steady-state conditions (flow rate of 0.011 m s^−1^).

**Fig. 5:**
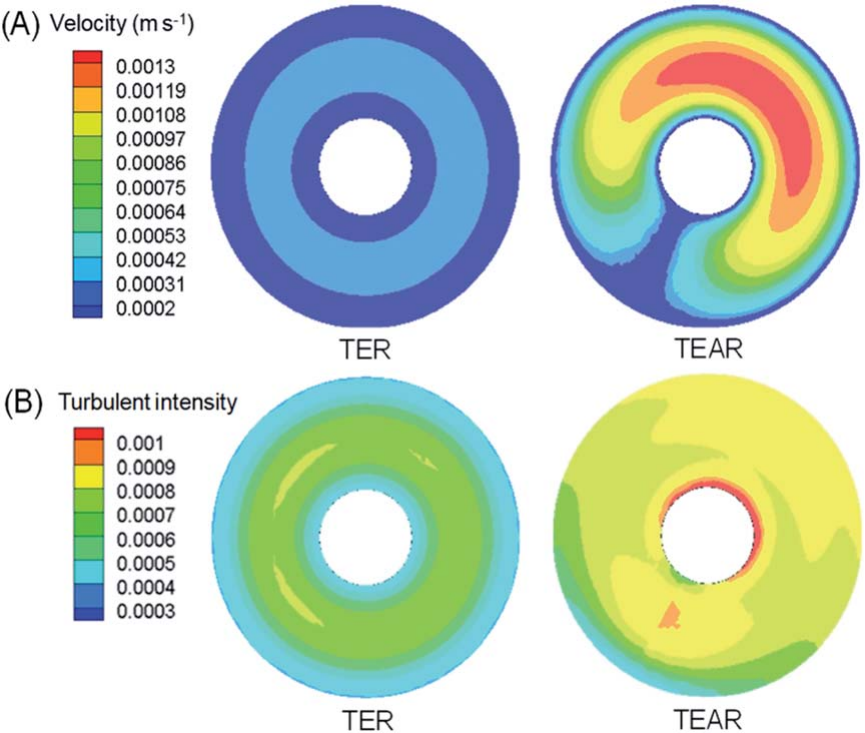
Vertical view of distribution of (A) velocity and (B) turbulent intensity in the TER and TEAR under steady-state conditions (flow rate of 0.011 m s^−1^).

**Fig. 6:**
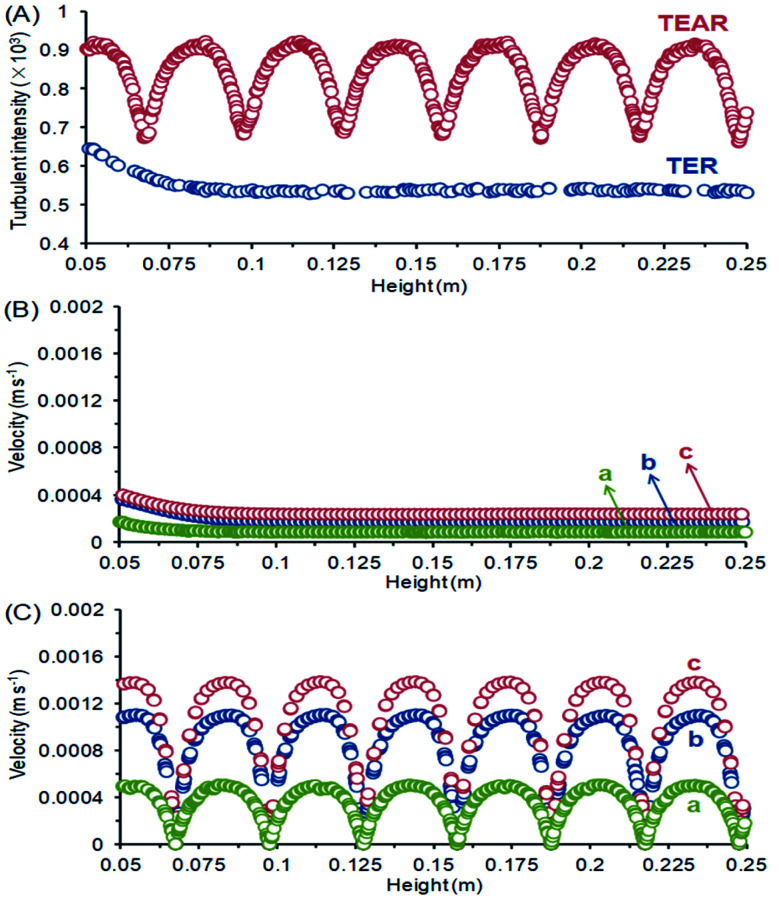
(A) Turbulent intensity, and velocity magnitude along the axial height of (B) TER and (C) TEAR at radial distances of (a) 15 mm, (b) 20 mm and (c) 30 mm.

The computational fluid dynamics (CFD) technology was used to simulate the distribution of flow fields. The velocity distribution is demonstrated in Fig. 4–6 by contrasting the reactor with static mixer and without. As shown, the axial face of the reactor showed a totally different flow field of the TEAR and tubular electrochemical reactor without mixer (TER), which was mainly caused by the spiral turbulence-promoting mixer. The reaction area was divided into a narrow and spiral channel with the mixer, causing a quite uniform flow velocity distribution with the maximum velocity nearly 0.15 cm s^−1^ compared to that without mixer (<0.03 cm s^−1^) (Fig. 4A). The turbulent intensity of the TEAR was larger than that of the TER, which revealed that the diffusion efficiency was strengthened by the mixer. The velocity distribution on radial direction was the central-symmetry of the TER; however, the TEAR was axial-symmetry by the section of mixer. The turbulent intensity distribution presented nearly the same characteristic with the velocity distribution, as well (Fig. 5). Besides that, the velocity and turbulent intensity were both enhanced by the application of the mixer. Turbulent intensity and velocity magnitude along the axial height are shown in Fig. 6. The turbulent intensity and the velocity magnitude present periodic distribution in the TEAR, while both of them decreased gradually along the axial height. The maximum of turbulent intensity in the TEAR was 0.9 × 10^−3^ while the minimum was 0.7 × 10^−3^, both larger than that of TER (Fig. 6A). The maximum of velocity magnitude in the TEAR was also larger than that of the TER; however, the minimum was 0 of the velocity magnitude along the axial height because of the spiral channel with the mixer. At a radial distance of 15 mm, the maximum of velocity magnitude in the TEAR was 0.0014 m s^−1^, while that was 0.0004 m s^−1^ in the TER. With the increase in velocity magnitude, the contact between the organic pollutants and the electrode would be reinforced, especially in the area with large axial height, resulting in the improved reaction rate.

### Stress and temperature enhancement of the TEAR

Oxygen evolution is the side-effect on the process of the anode electrode reaction.^41^ The oxygen produced by the side-effects could precipitate out in the form of bubbles, which would be covered on the electrode surface, thus isolating the contact between the electrolyte and the electrode surface, reducing the active site of the anode surface and the treatment efficiency of the reactor. The flow characteristics are the key factor of the bubble distribution on the electrode surface. Increasing the electrolyte flow can accelerate the undesired bubbles (side reactions) departing from the electrode surface by enhancing the wall shear force on the electrode surface. Therefore, the influence of the flow velocity on the bubble attachment on the electrode surface can be analyzed by comparing the shear stress on the electrode surface.

**Fig. 7:**
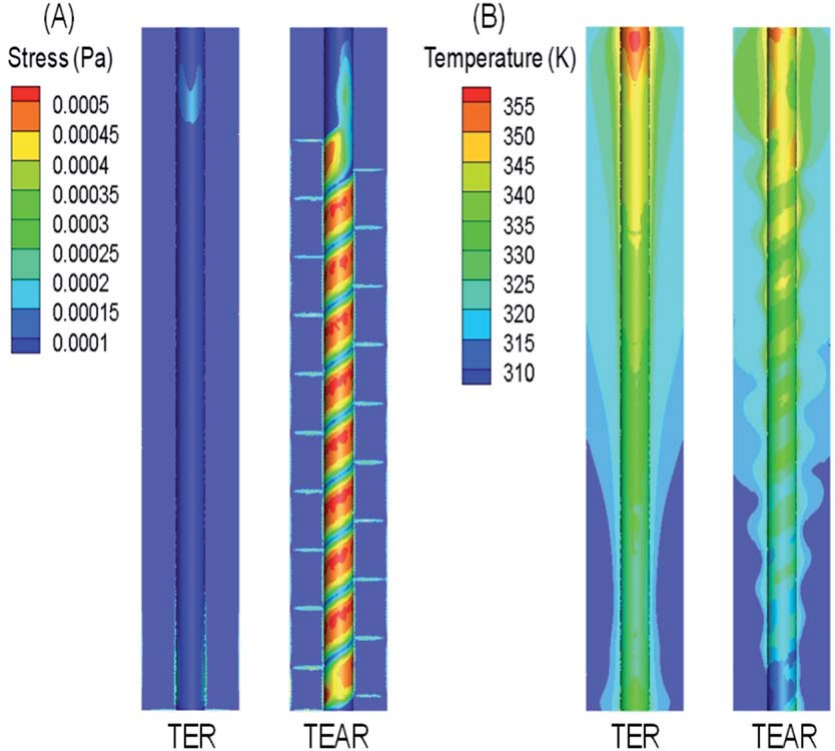
Front view of distribution of (A) surface stress and (B) temperature in the TER and TEAR under steady-state conditions (flow rate of 0.011 m s^−1^).

The shear force on anode surfaces and temperature field are depicted in Fig. 7. The shear force is in the range of 5.8–5.9 × 10^−6^ Pa for the TEAR, which was 4 times that of the reactor without any mixer (1.6–2.1 × 10^−6^ Pa) (Fig. 7A). The distribution of stress was similar to that of the velocity magnitude. In the TEAR, between the two layers of the spiral static mixer, shear stress was increased on the anode surface closer to the middle area, while in the area close to the mixer, because of a certain shear stress was applied by the mixer, the flow velocity was lower, and the shear stress was decreased. However, the shear stress was homogeneous in the TER.

Moreover, it is necessary to transfer heat from the reactor to the outside to keep the temperature at the desired set point. Pei^42^ indicated that at 70.2 °C, the concentration of the ˙OH radical used for removing organic pollutants was much lower than the concentration at 25 °C. Panizza^43^ pointed out that the current efficiency of the reactor was significantly improved when the temperature in the reactor was reduced from 60 °C to 20 °C. The temperature fields of both reactors were greatly consistent with their velocity fields, in which the temperature field distribution in the TEAR was more uneven (Fig. 7B), indicating that the spiral mixer can efficiently accelerate heat transfer. From the above-mentioned analyses, the induction of then static mixer was supposed to efficiently remove the bubbles instantly, weaken the electrode contamination, and strengthen the mass and heat transfer, resulting in a fast reaction of ˙OH radicals with the organic pollutants.

### Mass transfer coefficient enhancement of the TEAR

The mass transfer coefficient and Sherwood Number are the key factors to describe the mass transfer and concentration diffusion in the reactor. The relationship between mass transfer coefficient and Sherwood Number is *k*_*m*_ = Sh·*D*/*d*_e_. The Sherwood number can be obtained using eqn (4) and (5). Furthermore, the Reynolds number is Re = *ρvd*/*μ*, where *D* is the diffusion coefficient of the electrolyte, *d*_e_ is the hydraulic diameter of the tubular reactor and *d* is the pipe diameter of the reactor. The Schmidt number is Sc = *μ*/*ρD*, which is 272.7 in this research.

Laminar^44^

4 Sh = 0.62Re^0.5^·Sc^0.33^

Turbulent^45^

5 Sh = 0.0791Re^0.7^·Sc^0.356^

**Fig. 8:**
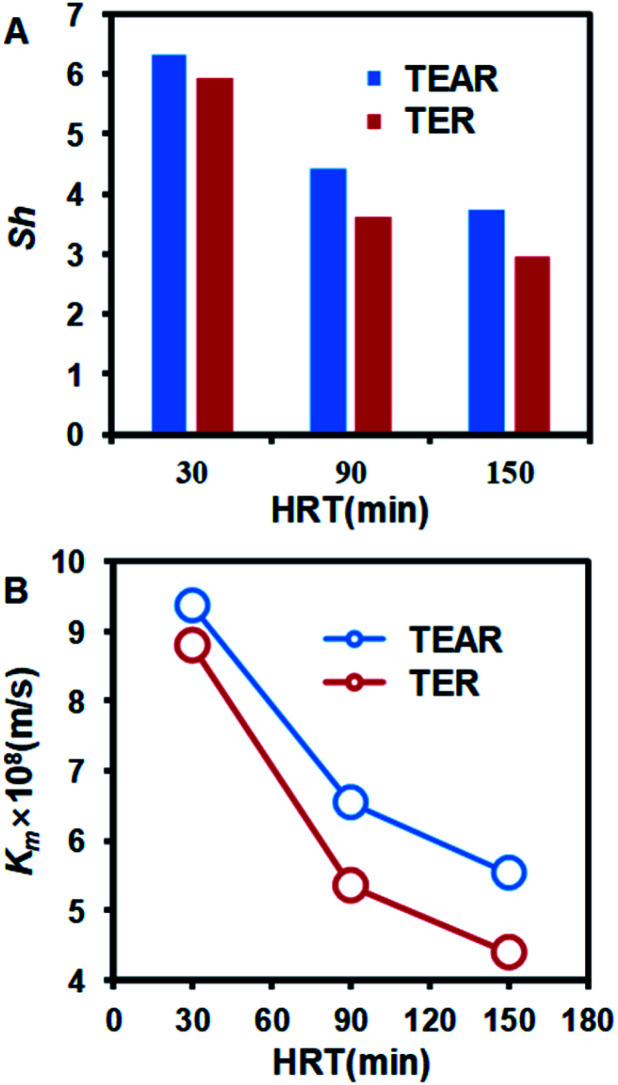
Comparison of (A) Sherwood number (Sh) and (B) mass transfer coefficient (*K*_*m*_) between the TER and TEAR.

The *k*_*m*_ and Sh number were compared between the TER and the TEAR, as shown in Fig. 8. Both of them were promoted by the application of the spiral static mixer. For different HRTs, the mass transfer coefficient and Sherwood number of the TEAR were nearly 1.2 times that of the TER. The reaction of EO occurred on the surface of the anode. In the process of EO, the reaction rate of EO is reaction control at a lower current density; however, at a higher current density, the reaction rate is mass transfer control. With the development of the mass transfer coefficient, the contact of the pollutant with the anode would be enhanced, which revealed that the baffle plate could be used in the pipelines for the construction of the tubular reactor and disposal of the industrial wastewater in the process of discharge.

## Conclusions

The novel tubular electrochemical assembly reactor (TEAR) can clearly enhance the wastewater treatment efficiency with an M-TiSO anode, an SSP cathode and a static mixer, which were all employed by *in situ* utilization. The M-TiSO is a kind of material that is stable and conductive, and the long-term stability was tested, inferring that M-TiSOs could be used as anodes, which are superior for efficient MB removal. The effect of the current density was discussed revealing the removal rate optimized at 9 mA cm^−2^ with a resident time of 90 min. The influence of hydrodynamic conditions was analyzed by CFD simulation and experiment, and the enhancement of mass transfer and reaction rate was demonstrated.

## Author contributions

J. L. performed conceptualization, methodology, data curation, simulation, writing review and editing. S. Y. performed conceptualization, supervision, validation, experiment, and writing original draft. YX. Y. performed supervision, validation, writing-review and editing. Y. Y. performed data curation. All authors approved the final version.

## Conflicts of interest

There are no conflicts to declare.

## Symbols and abbreviations

*ρ*
: Density of liquid

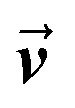

Velocity

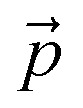

Pressure

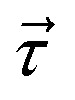

Stress tensor
*h*
: Sensible enthalpy
*λ*
: Thermal conductivity
*Φ*
: Viscous heat of liquid
*S*_h_
: Volumetric heat source
*T*
: Temperature

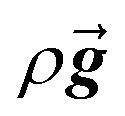

Gravitation body force

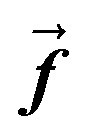

External force vector
Sh: Sherwood number
Re: Reynolds number
*k*_*m*_
: Mass transfer coefficient
Sc: Schmidt number
*d*
: Pipe diameter of the reactor
*d*_e_
: Hydraulic diameter of the reactor
*μ*
: Dynamic viscosity
*D*
: Diffusion coefficient
*C*
: Concentration of MB
*C*_0_
: Initial concentration of MB

## Supplementary Material

RA-011-D1RA02236A-s001
