# Supplementary material for: A tubular electrode assembly reactor for enhanced electrochemical wastewater treatment with a Magnéli-phase titanium suboxide (M-TiSO) anode and in situ utilization
Source: RSC Adv. 2021 Jul 20;11(40):24976–84. doi: 10.1039/d1ra02236a (PMC9036886; doi:10.1039/d1ra02236a)
Supplement: RA-011-D1RA02236A-s001 [file RA-011-D1RA02236A-s001.pdf]

Electronic Supplementary Material (ESI) for RSC Advances.
This journal is © The Royal Society of Chemistry 2021

Supporting Information

A Tubular Electrode Assembly Reactor for Enhanced Electrochemical Wastewater Treatment with Magnéli-phase Titanium Suboxides (M-TiSOs) Anode and *In-Situ* Utilization

Jiabin Liang*, Shijie You, Yixing Yuan, Yuan Yuan

State Key Laboratory of Urban Water Resource and Environment, School of Environment, Harbin Institute of Technology, Harbin 150090, P. R. China.

P. O. Box 2603#, No. 73, Huanghe Road, Nangang District, Harbin, 150090, China.

Tel.: +86−451−86282008; Fax: +86−451−86282110

E−mail: liangjiabin1234@foxmail.com (JB Liang)

Table of Contents

Additional Details on Methods

Table S1. Physical properties of electrolytes

Table S2. Treatment of industrial dyeing and finishing wastewater

Figure S1. The degradation rate of methylene blue (MB) by electrochemical oxidation at different current densities at HRT=90min

Figure S2. The ln(C/C0)-t diagram of methylene blue (MB) by electrochemical oxidation at different current densities at HRT=90 min

Figure S3. The degradation rate of TOC by electrochemical oxidation at different current densities at HRT=90min

Figure S4. The ln(C/C0)-t diagram of TOC by electrochemical oxidation at different current densities at HRT=90 min

Figure S5. The degradation rate of methylene blue (MB) by electrochemical oxidation at different hydraulic retention time (HRT) at current densities=8 mA/cm^2

Figure S6. The ln(C/C0)-t diagram of methylene blue (MB) by electrochemical oxidation at different hydraulic retention time (HRT) at current densities=8 mA/cm^2

Figure S7. The degradation rate of TOC by electrochemical oxidation at different hydraulic retention time (HRT) at current densities=8 mA/cm^2

Figure S8. The ln(C/C0)-t diagram of TOC by electrochemical oxidation at different hydraulic retention time (HRT) at current densities=8 mA/cm^2

Figure S9. The Comparison of electrochemical oxidation effect of methylene blue (MB) between TER and TEAR a) HRT=30 min; b) HRT=90 min; c) HRT=150 min

Figure S10. The Comparison of ln(C/C0)-t diagram of electrochemical oxidation effect of methylene blue (MB) between TER and TEAR a) HRT=30 min; b) HRT=90 min; c) HRT=150 min

Figure S11. The periodic CV tests at various scan rates for M-TiSOs electrode

Figure S12. SEM image of the surface of reacted TiSOs anode

Figure S13. The decolorization of real industrial wastewater

Table S1 Physical properties of electrolytes

Physical properties	Value
Temperature(*T*)	298k
Fluid density(ρ)	1095kg/m^3
Dynamic viscosity(μ)	2.12×10^{-4} Pa·s
Initial concentration (C_0)	50mg/L
Diffusion coefficient (D)	7.1×10^{-10} m^2/s
Schmidt number ($Sc=\mu/\rho D$)	272.7

Table S2 Treatment of industrial dyeing and finishing wastewater

Parameter	Before treatment	After treatment
sCOD (mg L^{-1})	155.3	78.6
Color (Pt-Co)	70	20
BOD_5/sCOD	0.088	0.153
pH	7.38	7.44

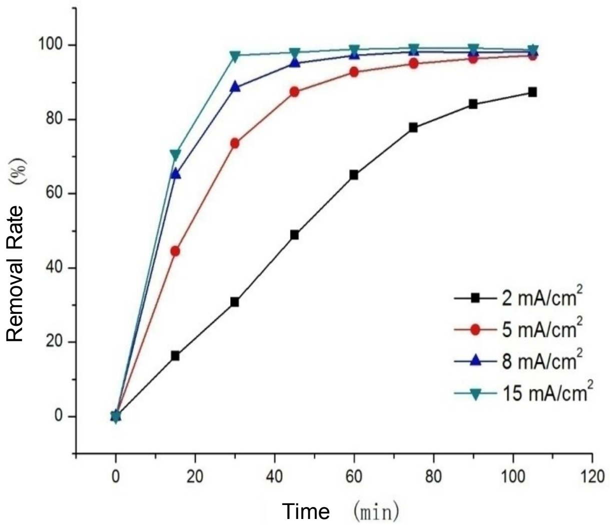

Figure S1. The degradation rate of methylene blue (MB) by electrochemical oxidation at different current densities at HRT=90min

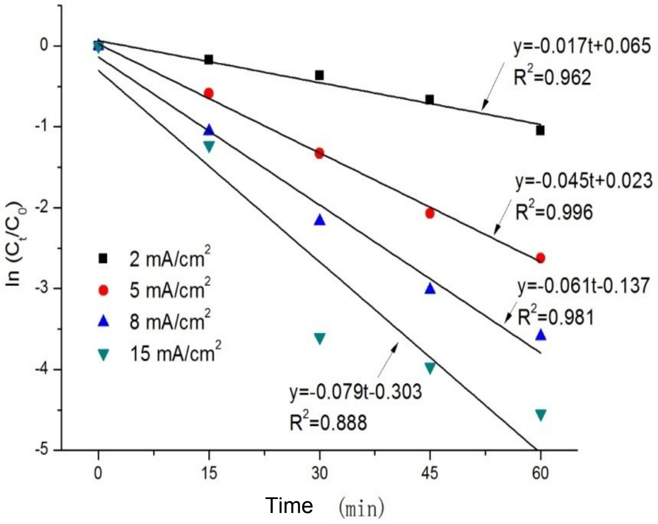

Figure S2. The ln(C/C_0)-t diagram of methylene blue (MB) by electrochemical oxidation at different current densities at HRT=90 min

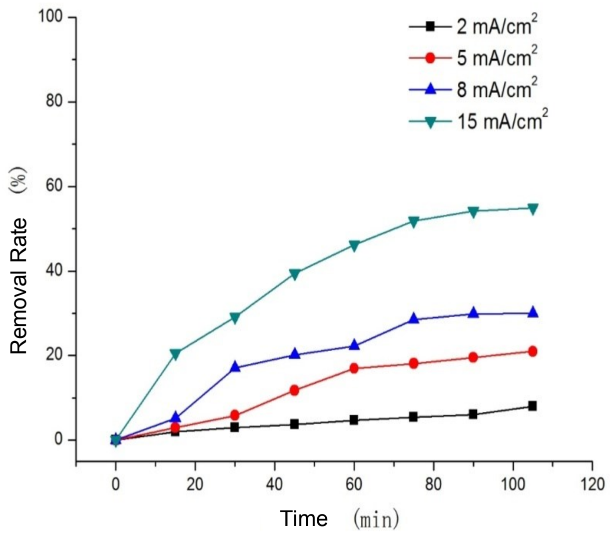

Figure S3. The degradation rate of TOC by electrochemical oxidation at different current densities at HRT=90min

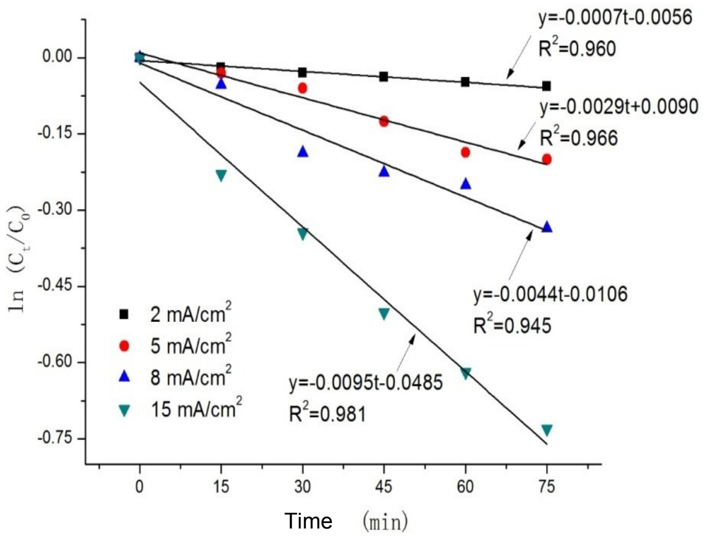

Figure S4. The ln(C/C_0)-t diagram of TOC by electrochemical oxidation at different current densities at HRT=90 min

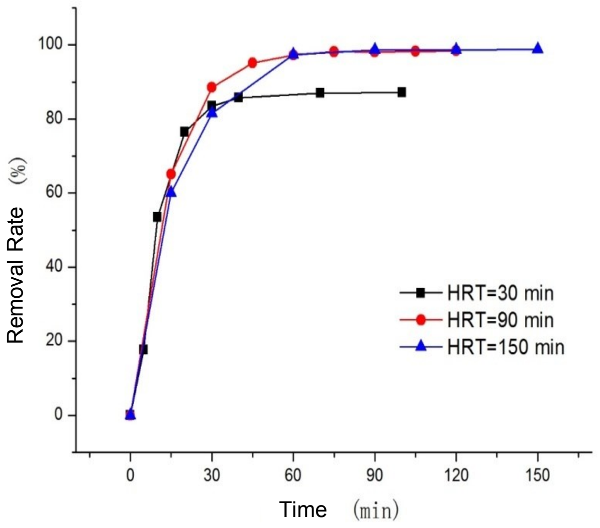

Figure S5. The degradation rate of methylene blue (MB) by electrochemical oxidation at different hydraulic retention time (HRT) at current densities=8 mA/cm^2

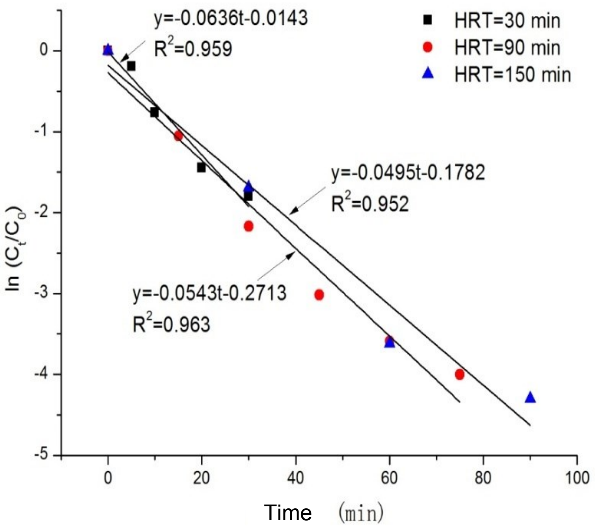

Figure S6. The ln(C/C$_0$)-t diagram of methylene blue (MB) by electrochemical oxidation at different hydraulic retention time (HRT) at current densities=8 mA/cm^2

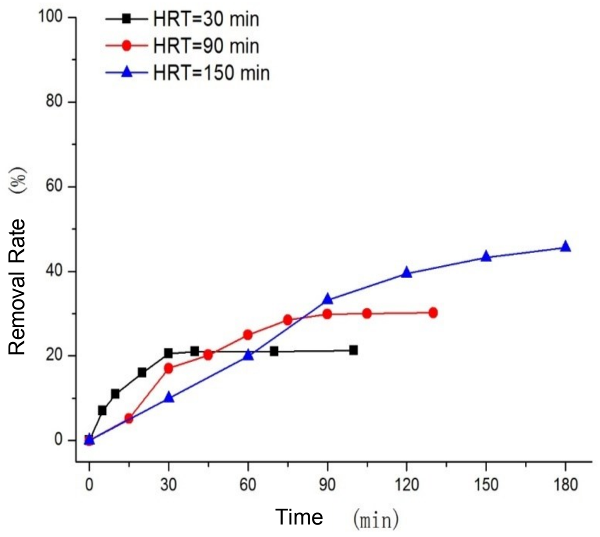

Figure S7. The degradation rate of TOC by electrochemical oxidation at different hydraulic retention time (HRT) at current densities=8 mA/cm^2

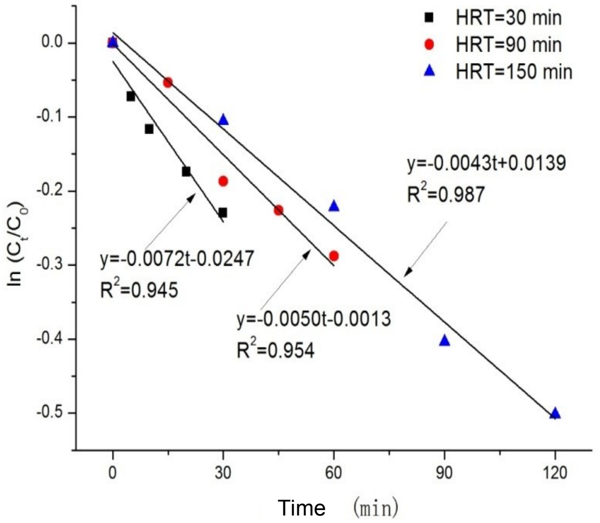

Figure S8. The ln(C/C$_0$)-t diagram of TOC by electrochemical oxidation at different hydraulic retention time (HRT) at current densities=8 mA/cm^2

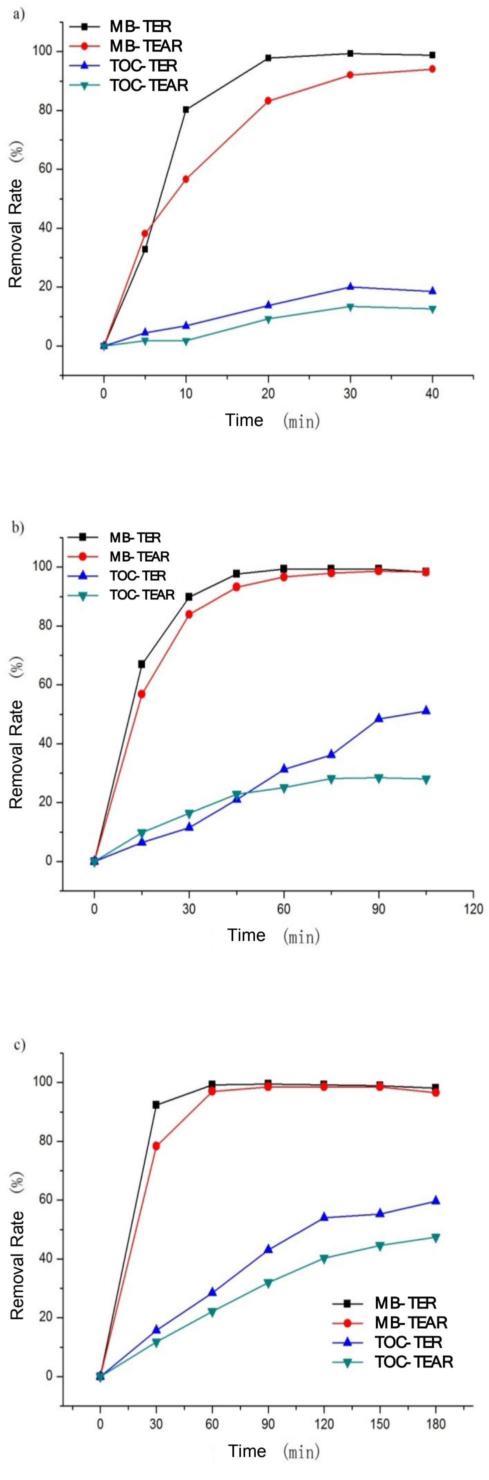

Figure S9. The Comparison of electrochemical oxidation effect of methylene blue (MB) between TER and TEAR a) HRT=30 min; b) HRT=90 min; c) HRT=150 min

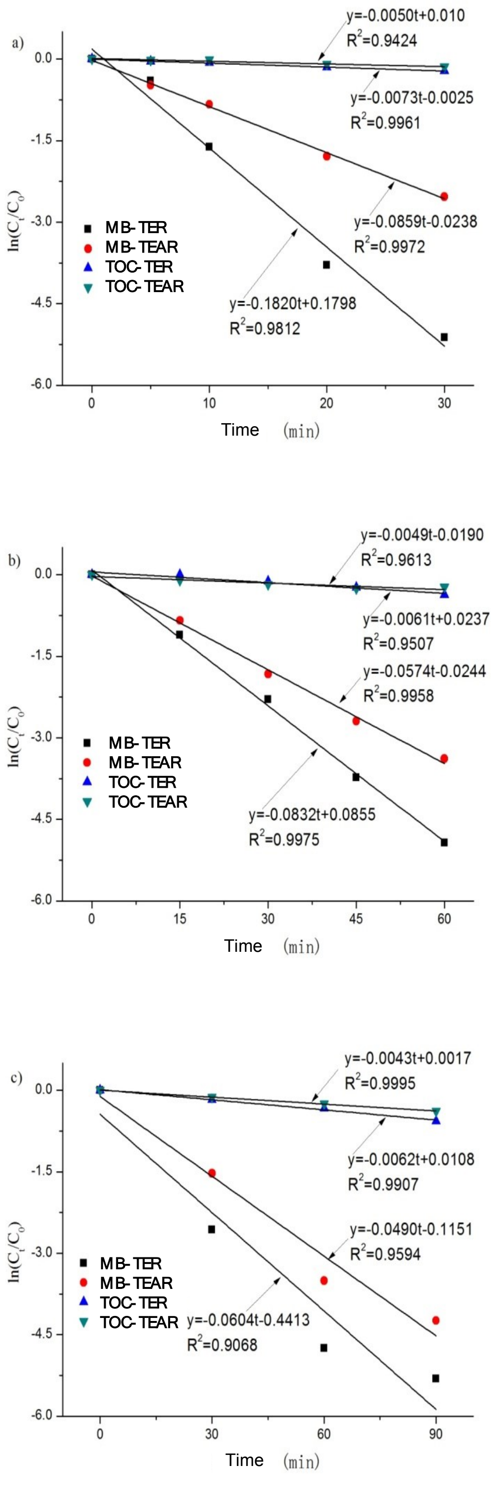

Figure S10. The Comparison of ln(C/C_0)-t diagram of electrochemical oxidation effect of methylene blue (MB) between TER and TEAR a) HRT=30 min; b) HRT=90 min; c) HRT=150 min

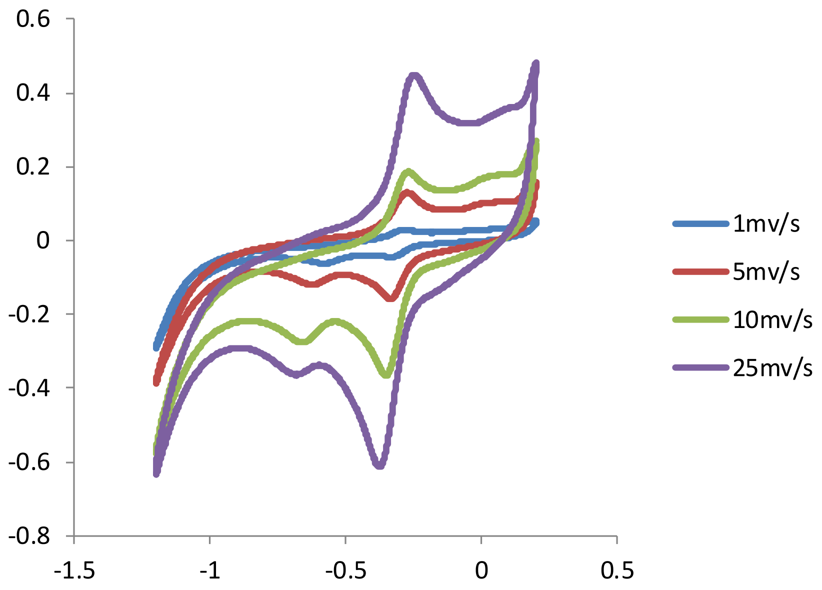

Figure S11. The periodic CV tests at various scan rates for M-TiSOs electrode

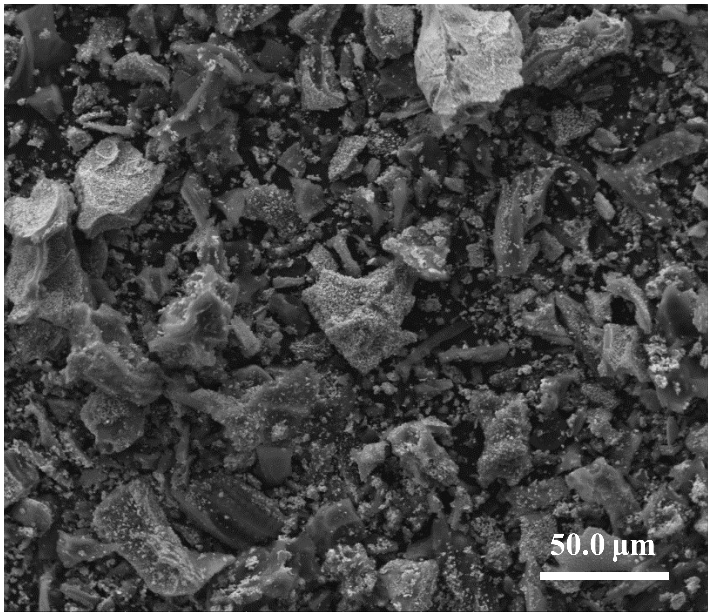

Figure S12. SEM image of the surface of reacted TiSOs anode

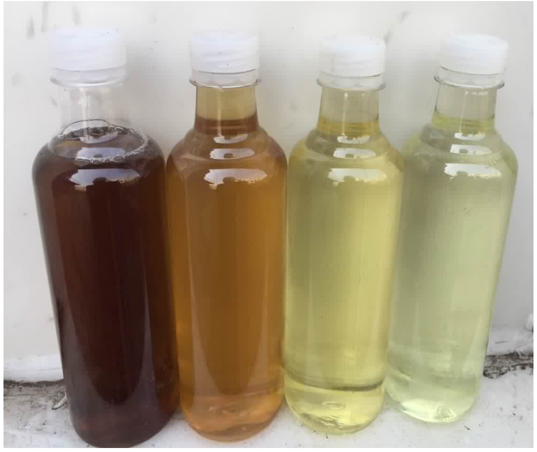

Figure S13. The decolorization of real industrial wastewater
